# Extensive-stage small cell lung cancer in Hungary: a real-world analysis (2013–2022)

**DOI:** 10.3389/pore.2026.1612491

**Published:** 2026-07-16

**Authors:** Krisztina Bogos, András Bajcsay, Gabriella Gálffy, Lilla Tamási, Zsolt Pápai-Székely, Veronika Sárosi, Anikó Maráz, Regina Pálföldi, Lilla Horváth, Judit Hoffer, Szilvia Szécsényi, Kata Kirschner, Celia Blasszauer, Dániel Reibl, Ida Komka, Anna A. Ascsillán, Lajos V. Kemény, Gyula Ostoros

**Affiliations:** 1 National Korányi Institute of Pulmonology, Budapest, Hungary; 2 Center of Radiotherapy, National Institute of Oncology, Budapest, Hungary; 3 Pulmonology Center of the Reformed Church in Hungary, Törökbálint, Hungary; 4 Károli Gáspár University of the Reformed Church in Hungary, Budapest, Hungary; 5 Department of Pulmonology, Semmelweis University, Budapest, Hungary; 6 Fejér County Szent György University Teaching Hospital, Székesfehérvár, Hungary; 7 Faculty of Medicine, University of Pécs, Pécs, Hungary; 8 Department of Oncotherapy, University of Szeged, Szeged, Hungary; 9 Department of Pulmonology, University of Szeged, Szeged, Hungary; 10 AstraZeneca Ltd., Budapest, Hungary; 11 MedicalScan Ltd., Budapest, Hungary; 12 HCEMM-SU Translational Dermatology Research Group, Semmelweis University, Budapest, Hungary; 13 Division of Infection and Immunity, University College London, London, United Kingdom

**Keywords:** epidemiology, etoposide-platinum, extensive-stage, overall survival, small cell lung cancer (SCLC)

## Abstract

This study provides a comprehensive nationwide analysis of extensive-stage small cell lung cancer (ES-SCLC) in Hungary, examining incidence rates, demographic trends, treatment patterns, and survival outcomes. We used data from the National Health Insurance Fund (NHIF) covering the period of 2013–2022, and we analyzed 8,104 ES-SCLC patients who received first-line (1L) etoposide-platinum (EP) chemotherapy, all of whom were confirmed to not have received concomitant chemoradiotherapy or curative thoracic surgery and had histology results in line with SCLC. We evaluated epidemiology, regional distribution, radiotherapy use, and subsequent treatment pathways. For the efficacy analysis, we narrowed the cohort to 5,576 patients who initiated EP within 1 year of their first C34-coded lung cancer diagnosis between 2013 and 2019, enabling 3-year follow-up. Key endpoints included overall survival (OS) and progression-free survival (PFS), the latter of which was inferred using time to first subsequent therapy (TFST). Our results revealed a shifting age distribution toward the age group above 70 years, while the male-to-female ratio gradually evened out. Treatment patterns showed the increasing use of carboplatin over cisplatin and frequent short course radiotherapy. Among those who underwent subsequent therapy, EP rechallenge was most widespread. Despite high initial response rates, survival outcomes remained poor: median PFS was 6.5 months (6-month: 52.9%, 1-year: 19%, 3-year: 5.2%), and median OS was 9.3 months (6-month: 69.8%, 1-year: 37.5%, 3-year: 9.2%). These results were in line with international real-world evidence and clinical trial data. Our findings highlight the aggressive nature of ES-SCLC and provide insight to the limited efficacy of chemotherapy-based therapies, underscoring the need to improve existing 1L and subsequent-line treatments and to introduce novel options. As Hungary transitions into the immunotherapy era, future studies with extended follow-up that incorporate staging, radiotherapy intent, comorbidities, and progression data will be essential for optimizing therapeutic strategies.

## Objective

The objective of this study was to determine overall survival (OS) and progression-free survival (PFS) in patients with extensive-stage small cell lung cancer (ES-SCLC) treated with first-line etoposide-platinum (EP) chemotherapy in Hungary from 2013 to 2019, using time to first subsequent treatment (TFST) as a proxy for PFS. TFST was used to infer PFS given that the National Health Insurance Fund (NHIF) database does not record PFS directly. Descriptive statistics were also prepared of patient demographics, regional incidence, and post-progression treatment strategies for the broader cohort diagnosed between 2013 and 2022.

## Introduction

Lung cancer remains the most frequently diagnosed cancer and the cause of cancer-related death worldwide [1–3], although in 2020 female breast cancer surpassed lung cancer in terms of new diagnoses (11.7% vs. 11.4%) but not mortality. While Hungary faced high lung cancer incidence and mortality throughout history [4–7], recent data suggest that the lung cancer burden has started to approach European averages [6, 8–11]. Among lung cancer subtypes, small cell lung cancer (SCLC) accounts for approximately 15%–20% of cases and is characterized by rapid progression and early metastasis, along with a strong association with smoking [12, 13]. Two thirds of all SCLC cases are classified as extensive-stage disease (ES-SCLC), which has a very poor prognosis [12, 13].

For more than two decades, first-line (1L) etoposide-platinum (EP) chemotherapy has been the standard of care in ES-SCLC [14], effectively inducing responses but producing only a limited survival benefit: median overall survival (OS) ranges between 8 and 10 months with this treatment [12, 15, 16]. In ES-SCLC, patients may receive non-curative dosages of thoracic and cranial radiotherapy as adjuvant therapy to complement chemotherapy, and ES-SCLC standard-of-care includes prophylactic cranial irradiation (PCI) too. Recently, phase III trials have introduced immunotherapy combinations (durvalumab-EP in CASPIAN, atezolizumab-EP in IMpower133) that yield improved patient outcomes compared with just EP [16, 17]. In Hungary, access to these therapies was limited to individual requests from 2022, until in 2024 they became nationally reimbursed [7].

Unlike in operable non-small cell lung cancer (NSCLC), where real-world data specific to Hungary are available [18], there is a lack of publications that characterize the Hungarian ES-SCLC population within the breadth of LC data, focusing on the treatment landscape and epidemiology [6]. We used the National Health Insurance Fund (NHIF) database to address this gap, which collects records of diagnoses, medical interventions, and clinical outcomes, covering nearly the entire population of Hungary [7].

In this real-world study, we analyzed patients who received 1L EP for ES-SCLC between 2013 and 2022, with an emphasis on epidemiology, treatment pathways, and survival data. PFS was inferred using TFST, and OS was determined in patient cohorts that were treated within 1 year of C34-coded diagnosis and had a minimum 3-year follow-up period following treatment, hence patients between 2013 and 2019. In sensitivity analyses, we excluded patients who received long-course (5 cycles or more, 25–30 fractions) of radiotherapy to reduce multimodal therapy bias, and kept those who underwent short-course therapy (1-2 cycles, 5–19 fractions maximum). Together these data form a national benchmark for assessing ES-SCLC outcomes in Hungary preceding the widespread implementation of immunotherapy [7].

## Materials and methods

### Data source

This nationwide, retrospective, longitudinal study was conducted using data from the NHIF. The NHIF is a national insurance system covering the Hungarian population almost entirely and collects patient IDs, reimbursed prescriptions, a wide range of interventions and events, and ICD-10-coded (International Statistical Classification of Diseases, version ICD-10) records of all domestic in-patient and out-patient visits. The NHIF also provides demographic data (age, gender, date of birth/death). For this study, we accessed the disease identification (ICD-10), diagnostics (DRG: Diagnosis Related Groups), and interventions (ICHI: International Classification of Health Interventions) sub-databases [7]. The NHIF includes a separate code only for concomitant chemoradiotherapy, not for sequential chemoradiotherapy. Furthermore, the NHIF does not include several variables that are relevant to clinical research, such as staging based on TNM classification, ECOG status, detailed histology for all patients, biomarker testing, laboratory results, adverse events, anamnestic history e.g., smoking, or therapies administered in clinical trials since they are not funded by the social security system [7]. All analyses were performed on anonymized records in alignment with national regulations and ethics approvals. The National Health Research Ethics Council approved the study (Reference: BM/15360–1/2023).

### Study population

Patients were eligible if they were newly diagnosed with lung cancer (ICD-10: C34) between 1 January 2013 and 31 December 2022 and were aged 18 years or older at diagnosis.

To specifically identify extensive-stage small cell lung cancer, we filtered for patients who received first-line systemic chemotherapy with a combination of etoposide and a platinum-based agent. We used the index year to mark the first EP administration and identified EP therapy with chemotherapy procedure codes (7092, 7097, 7188, 7189) and ICD codes concerning and related to the lungs (C34, C77, C78, C79). We excluded patients with codes and records that indicated thoracic surgery with curative intent (lobectomy, bilobectomy, pneumonectomy and variants, including sleeve resections and VATS anatomic resections) or concomitant chemoradiotherapy (cCRT: 7419, 7422, 7468) suggestive of radical intent, because these treatment patterns are associated with limited-stage SCLC [12]. Where available, tumor histology data was used to exclude patients with non-LC pathology, and those with LC histology definitively inconsistent with SCLC. This methodological approach was necessary because explicitly documented SCLC tumor histology was available for only 74.35% (6,026 of 8,104) of patients classified as having presumed extensive-stage SCLC based on treatment patterns, since reporting tumor histology to the NHIF is not mandatory when submitting therapy records.

Ideally, patients receiving either concomitant or sequential chemoradiotherapy would have been excluded in the first step of cohort selection. However, this was not possible, as the NHIF database provides a distinct code only for concomitant chemoradiotherapy, not for sequential chemoradiotherapy. Therefore, sequential chemoradiotherapy was assessed using a proxy definition in the sensitivity analysis.

For the epidemiological and treatment-pattern analyses, we used the full 2013–2022 ES-SCLC study cohort, and for the efficacy analysis, patients were selected if they started treatment between 2013 and 2019 to allow at least 3 years of follow-up. Subsequent therapy was defined as any new treatment administered after having completed 1L EP, and EP retreatment after a treatment-free interval of 3 months or more in the case of platinum sensitive patients, as patients treated with EP again within 3 months are platinum refractory patients.

**FIGURE 1:**
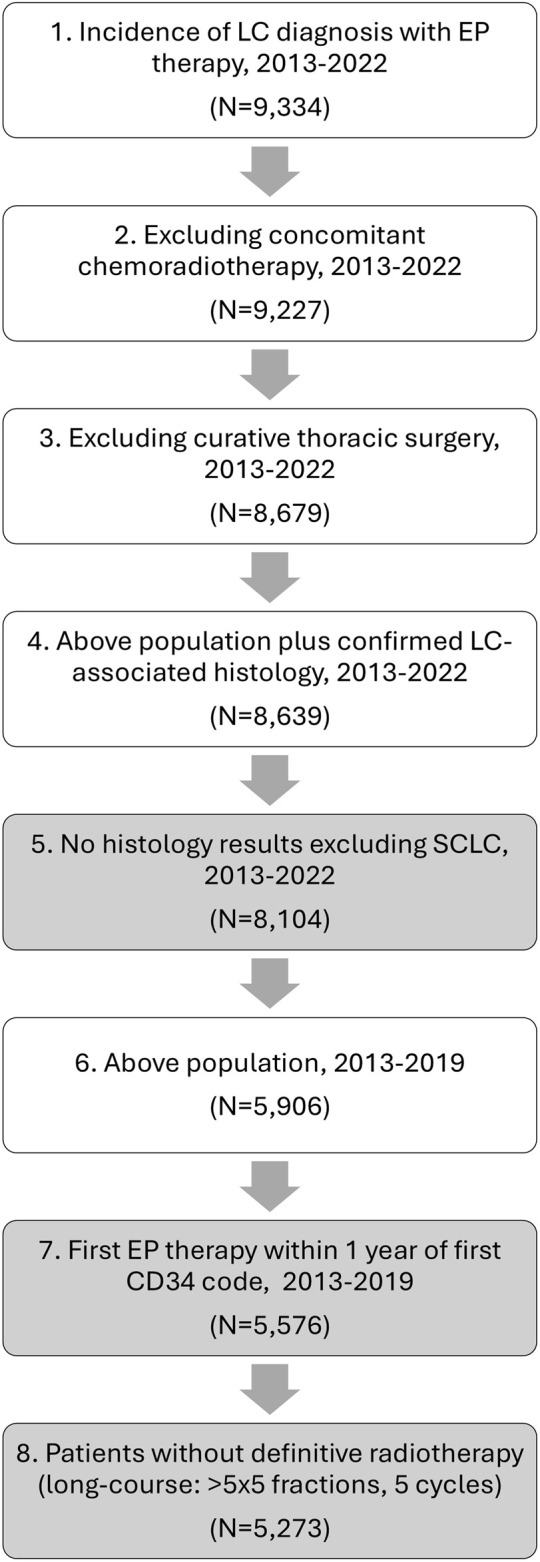
Illustration for the study inclusion/exclusion flow.

A sensitivity analysis re-estimated outcomes after removing patients who received long-course radiotherapy, reducing potential bias from multimodal therapy and preserving homogeneity across the cohort. We applied this method to indirectly exclude curative-intent sequential chemoradiotherapy, which is also associated with limited-stage SCLC, as it is missing from Hungarian coding practices. This outline was implemented cumulatively, with patient populations determined yearly (Figure 1):

1. Between 2013 and 2022, 9,334 patients diagnosed with lung cancer (ICD-10: C34) underwent etoposide-platinum therapy.
2. Patients treated with concomitant chemoradiotherapy were excluded, leaving 9,227 in the study cohort.
3. A total of 8,679 patients were then confirmed to have not received thoracic surgery with curative intent.
4. 8,639 patients were kept from the above cohort when histology results were consulted (upon availability) and found LC-associated diagnoses.
5. Of these, 8,104 had no histology results excluding SCLC and thus remained in the study cohort.
6. 5,906 patients had data available between 2013 and 2019, allowing for a 3-year follow-up period.
7. Finally, 5,576 patients had undergone treatment within 1 year of C34-coded diagnosis and constituted the PFS and OS analysis cohort.
8. From the above population, patients who underwent long-course radiotherapy were excluded for sensitivity analysis, reducing the cohort to 5,273 patients.

### Statistical analysis

To evaluate clinical outcomes in the ES-SCLC population, we analyzed overall survival (OS) and progression-free survival (PFS). In the NHIF database, OS can be determined reliably, but progression is not recorded, therefore, PFS could not be assessed directly. For this reason, time to first subsequent treatment (TFST) or death was used as a proxy for PFS. Although TFST is assumed to correlate with PFS, a patient’s condition may worsen without initiation of subsequent treatment, thereby prolonging TFST. This may lead to distortion of the true PFS, which cannot be quantified from the available data.

TFST was defined as the interval between the first administration of EP and the start of subsequent therapy or death. Patients who did not experience an event (initiation of subsequent therapy or death) were censored at the end of the study observation period (December 31, 2022). Since the NHIF database provides near-complete national coverage, traditional loss to follow-up is negligible.

We used R software (version 4.1.2), specifically ‘survival’ and ‘survminer’ packages for all Kaplan-Meier analyses.

Kaplan-Meier methodology helped estimate survival curves for both TFST and OS. This non-parametric statistic is useful for evaluating time-to-event data to estimate treatment durability, especially suited to analyze the time until a marked event such as death or disease relapse.

Linear regression models the relationship between a dependent and one or more independent variable and was used in this study to determine annual trends in patient numbers and other data per 100,000 people.

Among the point estimates and confidence intervals (CI) reported, Kaplan-Meier survival analysis indicated survival probability estimates. Linear regression gave insight into the direction and magnitude of patient trends over time. Adjusted and unadjusted estimates were both reported, the precision of which were denoted by 95% CI.

Descriptive statistics were also compiled based on age, gender, and geographic distribution i.e., by county and year of diagnosis, calculating incidence rates relative to the Hungarian population in 2022. Treatment patterns were summarized according to number of EP cycles, radiotherapy use, and subsequent therapy lines by number and types.

## Results

Epidemiological analysis was performed on 8,104 patients with ES-SCLC, while 5,576 patients who received 1L EP chemotherapy within 1 year of their initial C34 diagnosis were selected for our follow-up efficacy analysis 3 years after undergoing treatment. Of the 5,576 patients within the latter cohort, 5,286 (94.8%) either received first subsequent therapy or died, indicating disease progression, while 290 (5.2%) lived throughout the full study period without the administration of subsequent therapy. Of patients without radiotherapy or with short-course radiotherapy (N = 5,273), 5,009 (95%) either received subsequent therapy or died, while 264 (5%) stayed alive throughout the 3-year period.

### Cohort characteristics

**FIGURE 2:**
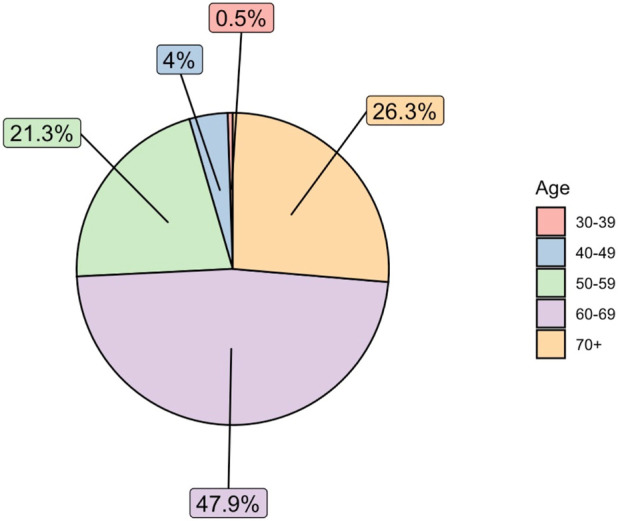
Average age distribution among patients over the study period between 2013 and 2022.

Among the 8,104 patients diagnosed with ES-SCLC and treated with 1L EP chemotherapy between 2013 and 2022, the majority, 47.9% (3,877/8,104), were aged 60–69 years at diagnosis (Figure 2). In 2013, 5.14% (43/836) of patients were aged below 50,28.2% (236/836) were between 50 and 59 years old, 44.6% (373/836) fell into the age range of 60–69 years, while 22% (184/836) were aged 70+. By 2022, we observed a gradual shift toward older age, as the proportion of patients under 50 years of age was 3.75% (27/720), 16.7% (120/720) were between 50 and 59 years, and the age group of 60–69 years reached 44.4% (320/720), meanwhile the 70+ group rose to 35.1% (253/720) (Supplementary Figure S1). These patterns align with previous national demographics separately reported for lung cancer [7, 10].

**FIGURE 3:**
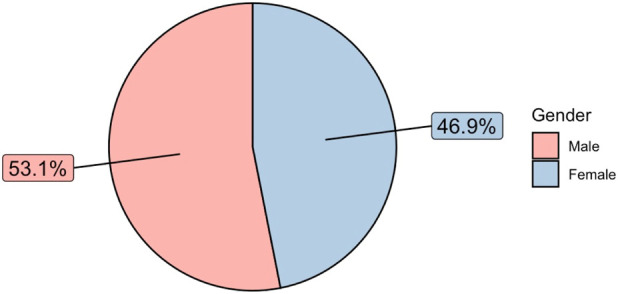
Average gender distribution among patients over the study period between 2013 and 2022.

In terms of gender, ES-SCLC was more commonly diagnosed in men, accounting for 53.1% of cases, than in women, representing 46.9% overall (Figure 3). However, this gap progressively narrowed over the study period: compared to the male-to-female ratio of 53.7% vs. 46.3% in 2013, the gender distribution in 2022 was 50.0% vs. 50.0% (Supplementary Figure S2).

**FIGURE 4:**
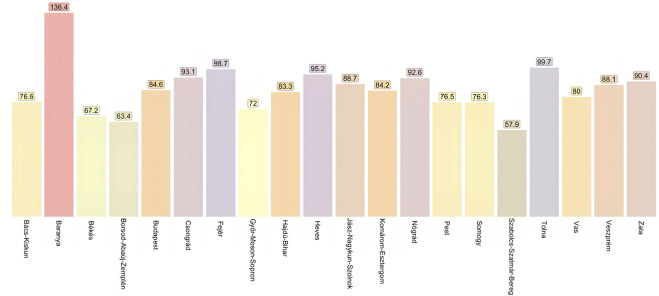
Distribution of new patients by county in the given year, compared to the KSH data (2022) per 100,000 inhabitants.

Regionally, ES-SCLC incidence varied by county. Using the 2022 county population data from the Central Statistical Office (KSH) as a reference, the highest incidence was observed in Baranya County with 136.4 per 100,000 (95% CI: 124.4–148.4). This was notably above the national average of 82.7 per 100,000 (95% CI: 80.9–84.5), while numerous other counties were proximal to the national mean [7] (Figure 4).

### Treatment patterns

As per our study design, ES-SCLC patients received 1L EP chemotherapy, with or without non-curative thoracic and/or brain radiotherapy or prophylactic cranial irradiation administered according to standard-of-care ESMO and NCCN guidelines [12, 19]. Any therapies that were given as part of clinical trials are unknown to us given that the NHIF database does not record them, as such they fall outside the scope of our analysis.

Between 2013 and 2022, in the full ES-SCLC cohort the annual number of EP-treated lung cancer patients went down from 836 in 2013 to 720 in 2022 (index year determined by the first EP treatment). In the full ES-SCLC cohort (N = 8,104), most patients were administered carboplatin-based EP (carboplatin/cisplatin ratio 71% vs. 29%) between 2013 and 2022, whereas the use of cisplatin diminished gradually over time (carboplatin/cisplatin ratio between 2013 and 2022: in 2013, 63% vs. 37% and in 2022, 87% vs. 13%). In terms of treatment intensity, we saw that 46% (3,756/8,104) of patients, the majority, completed two to four cycles of EP, 34% (2,752/8,104) underwent 5 or more cycles, and 20% (1,594/8,104) received a single cycle during 1L therapy.

38.4% (3124/8,104) of the cohort underwent radiotherapy at some point during their treatment, most commonly this amounted to a short course, as in 33.7% (2742/8,104) of the patients. Only 4.7% (382/8,104) of the patients received long-course thoracic radiotherapy, and as mentioned before, given that higher cumulative radiotherapy treatments reflect therapeutic pathways with potential curative intent, we also analyzed outcomes in sensitivity analyses excluding this subgroup to focus on chemotherapy-centered ES-SCLC treatment.

Subsequent therapy was frequent among the 2013–2022 cohort (N = 8,104), with 38.7% (3,139/8,104) receiving second-line therapy. The most widely used approach was EP retreatment with 49.7% of subsequent therapy patients receiving it, and for platinum refractory patients, cyclophosphamide-epirubicin-vincristine/etoposide combinations with 28.1%, followed by topotecan 17.9%, other platinum-based doublets 1.6%, and other agents 2.7%. Approximately 6% of patients in the full ES-SCLC cohort underwent more than two lines of treatments.

### Efficacy results

To evaluate clinical outcomes, we analyzed the data of 5,576 patients diagnosed with ES-SCLC who received 1L EP chemotherapy within 1 year of diagnosis between 2013 and 2019, allowing for a minimum follow-up of 3 years. Following a C34-coded diagnosis, years may pass before undertaking 1L EP chemotherapy. Therefore, this study distinguishes between patients who received 1L EP treatment within 1 year of initial C34 diagnosis (N = 5,576) and those who did not. We determined PFS, OS, Kaplan-Meier curves, median values, and proportions in our cohort at the aforementioned timepoints (PFS/OS: 6 months, 1 year, 2 years, 3 years).

The data listed above was determined in the following two groups of those who received 1L EP chemotherapy within 1 year of diagnosis: the first group consisted of patients recorded between 2013 and 2019 after initial C34-coded diagnosis and subsequent 1L chemotherapy treatment within 1 year of diagnosis (N = 5,576), and the second one contained the same patients, yet excluding those who underwent long-course radiotherapy (N = 5,273) (sequential chemoradiotherapy indicative of limited stage SCLC). This exclusion lowered both PFS and OS values.

### Progression-free survival (PFS)

**FIGURE 5:**
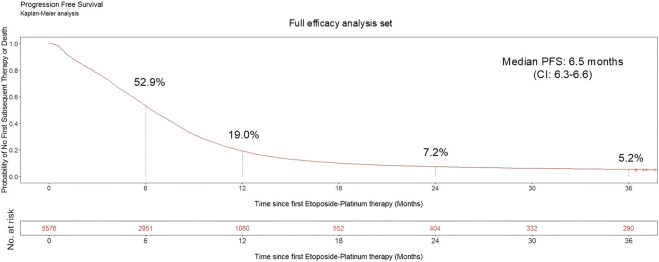
Kaplan-Meier curve of PFS (months) analysis between 2013 and 2019 (N = 5,576) of patients receiving treatment within 1 year of C34 diagnosis.

Progression is not recorded in the NHIF database. Therefore, PFS could not be assessed directly. For this reason, we used TFST or death as a proxy for PFS. The Kaplan-Meier curve of TFST between 2013 and 2019 (N = 5,576) showed a median PFS of 6.5 months, along with the 1-year PFS rate of 19% and a 3-year PFS rate of 5.2% (Figure 5).

The first 6 months proved to be critical, as only 52.9% of patients remained progression-free at that point (Figure 5). This observation is consistent with published real-world data and data from clinical trials on EP-treated ES-SCLC [16, 17].

When we excluded patients who received long-course radiotherapy (N = 303) from our sensitivity analysis, results derived from the remaining population (N = 5,273) showed a median PFS of 6.3 months along with a 6-month PFS rate of 51.8% and a 3-year PFS rate of 5.0%, further substantiating the robustness of the primary analysis (Supplementary Figure S3).

### Overall survival (OS)

**FIGURE 6:**
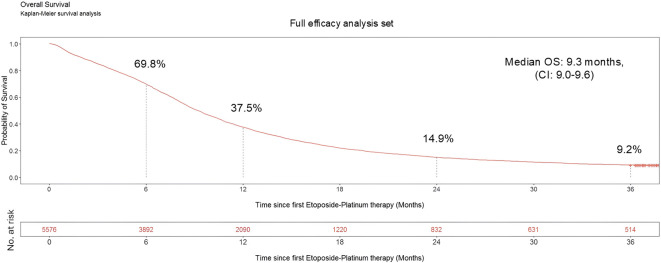
Kaplan-Meier curve of OS (months) analysis between 2013 and 2019 (N = 5,576) of patients receiving treatment within 1 year of C34 diagnosis.

Along with PFS, OS was also evaluated in the 2013–2019 patient cohort who were treated within 1 year of diagnosis (N = 5,576). The median OS was 9.3 months, with a 1-year OS rate of 37.5% and a 3-year OS rate of 9.2%. It is worth noting that 69.8% of the patients survived the first 6 months, and 14.9% were still alive after 2 years (Figure 6).

The OS analysis that excluded long-course radiotherapy cases (N = 5,273) confirmed the representative power of our primary population with a median OS of 8.9 months along with a 6-month PFS rate of 68.3% and a 3-year PFS rate of 8.7%, not showing any substantive difference from the inclusive analysis (Supplementary Figure S4).

## Discussion

It is worth reaffirming that this nationwide, retrospective real-world study followed ES-SCLC cases in Hungary from 2013 to 2022, with the sociodemographic, clinical characteristics, diagnostic, and treatment pattern analysis cohort consisting of 8,104 patients treated with 1L EP chemotherapy during that period. The efficacy analyses were performed on a tighter cohort of 5,576 patients treated between 2013 and 2019 within 1 year of their initial C34 diagnosis to allow for 3-year follow-up. Within this framework, we provide details on progression, and survival outcomes before the implementation of immunotherapy as a treatment option in 2024.

The incidence of ES-SCLC in Hungary cannot be determined directly, as no available national database contains the annual number of lung cancer diagnoses together with reliable histological results and TNM classification data. To address this gap, we developed a methodology to identify the ES-SCLC population using the NHIF claims database.

Although the NHIF database includes a wide range of treatments and interventions, only concurrent chemoradiotherapy has a distinct reimbursement code, whereas sequential chemoradiotherapy is not coded separately. If sequential chemoradiotherapy had been clearly identifiable in the coding system, patients receiving either concurrent or sequential chemoradiotherapy should have been excluded in the first step of cohort selection. Consequently, an important limitation of our study is that, as sequential chemoradiotherapy could not be directly identified, the exclusion criteria included only concurrent chemoradiotherapy, which may have resulted in a slight overestimation of the number of ES-SCLC patients.

To further assess the robustness of our findings, we conducted a sensitivity analysis using a proxy definition for sequential chemoradiotherapy. Specifically, we excluded patients who received long-course radiotherapy after EP chemotherapy, based on the coding practices reported by local oncoradiologists. However, this approach may have led to a slight underestimation of the total number of ES-SCLC patients, as the database does not allow differentiation between patients who received radiotherapy as part of sequential chemoradiotherapy with curative intent for limited-stage SCLC and those who received radiotherapy for palliative purposes after EP chemotherapy in the ES-SCLC setting. As no formal reference defining long-course radiotherapy by a specific dose threshold was identified, the threshold applied in this study was based on routine oncoradiological practice. This is consistent with international clinical practice, in which conventional once-daily thoracic radiotherapy for LS-SCLC commonly consists of 50–66 Gy delivered in 2-Gy fractions. Because radiotherapy is recorded weekly in the NHIF database, this schedule corresponds to 5 or more reported cycles, that is, approximately 25–30 fractions.

Our cohort is at present one of the largest national ES-SCLC datasets in Central Europe. EP chemotherapy patterns captured by the NHIF helped identify patients, and exclusion rules were set based on surgical and concomitant chemoradiotherapy codes characteristic of limited-stage SCLC or curative-intent treatment. The patient population reflects the known demographics of ES-SCLC: men were more frequently affected than women (53.1% vs. 46.9%), and the gender gap slightly narrowed over time, paralleling the reports of shifting smoking trends across Central and Eastern Europe [10, 12, 14], as reported in the Korányi Bulletin and earlier NHIF analyses. Some years saw nearly equal male-to-female ratios (50.3% vs. 49.7% in 2020; 50% vs. 50% in 2022), The Korányi Bulletin recorded a shift in the total lung cancer population within the lung care system over the years: 84% vs. 16% in 1980, 61% vs. 39% in 2013, 53% vs. 47% in 2022.

The majority of cases occurred between ages 60–69 years, slightly shifting toward older ages with time: the proportion of patients aged 70–79 years increased, whereas the proportion of patients below 60 declined. This shift was more pronounced in other studies assessing the entire Hungarian lung cancer population [8]. Furthermore, a large Canadian retrospective RWE study (N = 1,941) of ES-SCLC reported that 81% of their cohort were over 60 [20]. Another study involving five European countries found that the median age of SCLC patients was 66 [21], both reporting similar data to ours.

We observed regional differences as well, with Baranya County reaching the highest age-standardized incidence at 136.4 per 100,000 (95% CI: 124.4–148.4), compared with the national average of 82.7 per 100,000 (95% CI: 80.9–84.5) based on KSH data. This geographic variation may reflect differences in underlying environmental or population-level risk factors. Given Baranya county’s mining history, including operation of a uranium mine between 1957 and 1997, it may be hypothetized that historical environmental exposures could have contributed to the observed pattern. Additionally, several studies have reported association between air pollution and SCLC risk [22–24]. However, these interpretations remain speculative, and no causal relationship can be established from the present data.

EP chemotherapy was the first-line treatment regimen throughout the study period [25, 26], wherein carboplatin use increased, gradually replacing cisplatin administration. Canadian and American studies showed that most patients received carboplatin rather than cisplatin as a platinum agent in 1L ES-SCLC therapy in recent years [20, 27]. In terms of treatment delivery, the course length fell between 2–4 cycles in roughly 46% of cases. Approximately 20% received a single cycle, and this halting of treatment likely reflects chemotherapy intolerance or rapid clinical deterioration in patients.

Long-course radiotherapy generally reflects curative-intent sequential chemoradiotherapy in limited-stage SCLC. In our cohort, more than a third (38.4%) of the patients received radiotherapy, but only small rate of them (4.7%) underwent long-course thoracic radiotherapy.

Among progressing patients, 38.7% received second-line therapy, with EP rechallenge making up almost half of those cases. According to standard European practices, EP rechallenge is preferred in platinum-sensitive relapsed cases. Access to later-line agents such as topotecan and ECO and ECO/B (cyclophosphamide, epirubicin, and vincristine) varied; among patients on subsequent treatment, 17.9% received topotecan and 28.1% received ECO and ECO/B. In international studies, topotecan use ranged from 9% to 40.8% [28, 29] under similar circumstances, and the 2013 ESMO guideline recommended intravenous topotecan and the classical CAV regimen consisting of cyclophosphamide, doxorubicin, and vincristine for platinum-refractory patients [14]. Although the guideline refers to CAV, in Hungarian clinical practice the corresponding regimens used in lung cancer are ECO and ECO/B, which include cyclophosphamide, epirubicin, and vincristine, in accordance with the local chemotherapy protocol [30].

This analysis confirms that ES-SCLC is an aggressive form of cancer that carries poor long-term survival outlooks: Both the directly measured OS and the TFST-proxied PFS paint a similar picture with quick progression emerging frequently despite initial treatment. The median PFS was 6.5 months, where only 52.9% and 19% of patients were progression-free at 6 months and 1 year, respectively, and 5.2% at 3 years. The median OS was 9.3 months, with a 1-year survival rate of 37.5%, and a 3-year survival rate of 9.2%. In the sensitivity analysis, when we excluded patients with long-course radiotherapy, we found similar results, with a median PFS of 6.3 months, a 6-month PFS rate of 51.8% and a 3-year PFS rate of 5.0%. The median OS in the sensitivity analysis was 8.9 months, with a 1-year survival rate of 35.6%, and a 3-year survival rate of 8.7%.

All these efficacy results are consistent with the outcomes reported in other European real-world studies preceding immunotherapy. France reported an OS of 11.8 months and PFS of 6.1 months, while in Spain OS was 8.2 months and PFS was 5 months [15, 16, 31].

In the CASPIAN study, a phase III randomized clinical trial investigating combination therapy with durvalumab and EP in ES-SCLC patients, the control EP arm reportedly had 12- and 24-month PFS rates of 5.3% and 2.9%, with a median PFS of 5.4 months. In IMpower133 study that involved atezolizumab administration in combination with EP, the EP control arm PFS figures were 22.4% at 6 months, 12% at 24 months, and the median PFS was 4.3 months [16, 17, 32].

The slightly longer PFS observed in our study can be attributed to differences in study methods, as progression was proxied using TFST. Given that ES-SCLC is a rapidly progressing disease, even short delays between radiologic progression and the initiation of subsequent therapy may extend TFST in relation to the trial-defined PFS. Furthermore, if supportive care was provided without follow-up therapy, this period was not captured.

Regarding the shorter OS found in our analysis, we propose that it may be due to the criteria of patient selection in clinical trials being much stricter than real-world patients’ data reflect (performance status 0–1, kidney and liver function, life expectancy at least 12 weeks, etc.).

## Conclusion

This nationwide real-world analysis characterizes the Hungarian ES-SCLC patient population in the pre-immunotherapy era, and it demonstrates survival outcomes that align with those reported in the EP control arms of the CASPIAN and IMpower133 trials. The slightly longer PFS observed in our study can be explained by PFS being proxied using TFST. However, the shorter OS reflects that real-world patients do not fit the strict eligibility criteria of phase III trials. Together, these findings confirm that the ES-SCLC cohort of Hungary behaves consistently with clinical expectations, and the minor deviations between outcomes are due to well-known differences between routine care and clinical trial frameworks.

Despite the limitations presented by the NHIF database (lacking safety or adverse events data), our study was able to outline the national ES-SCLC population, their treatment pathways and radiotherapy patterns. NHIF data would prove to be more widely applicable if it distinguished between sequential chemoradiotherapy and palliative or prophylactic radiotherapy. The lack of validated biomarkers in ES-SCLC further highlights the need for robust biomarker discovery and prospective validation studies to enable biologically guided patient selection and personalized therapeutic strategies. An ideal cancer registry would operate with standardized coding practices, record structured oncologic variables, and have improved granularity, aiding in determining progression timing and the reason for discontinued treatment.

Given the complexity of ES-SCLC management, multidisciplinary collaboration remains crucial: pulmonary oncologists, radiation oncologists, thoracic surgeons, radiologists, palliative care specialists, and data science analysis teams all elevate patient care and maintain the high quality of real-world evidence.

Immunotherapy is continuously adopted in practice across Hungary; thus it is important to note that the accumulation of real-world follow-up data of chemo-immunotherapy will take time. As of now, long-term data is only available for chemotherapy-based treatment. This renders the current analysis a reliable national baseline, also complementing international evidence as one of the largest ES-SCLC cohorts in Europe, for future comparison of outcomes and assessing the impact of chemo-immunotherapy in practice.

## Data Availability

Publicly available datasets were analyzed in this study. This data can be found here: Real-world data from the National Health Insurance Fund (NHIF) under the reference I043/49-4/2024, accessed by MedicalScan Ltd.

## References

[1] Bray F Soerjomataram Isabelle JF Siegel RL Torre LA Jemal A . Global cancer statistics 2018: GLOBOCAN estimates of incidence and mortality worldwide for 36 cancers in 185 countries. CA Cancer J Clin (2018) 68(Epub):394–424. 10.3322/caac.21492 30207593

[2] Sung H Ferlay J Siegel RL Laversanne M Soerjomataram I Jemal A Global cancer statistics 2020: GLOBOCAN estimates of incidence and mortality worldwide for 36 cancers in 185 countries. CA Cancer J Clin (2021) 71(3):209–49. 10.3322/caac.21660 33538338

[3] World Health Organization. Cancer fact sheets: lung cancer (2022). Available online at: https://www.who.int/news-room/fact-sheets/detail/lung-cancer (Accessed August 18, 2026).

[4] Ferlay J Steliarova-Foucher E Lortet-Tieulent J Rosso S Coebergh JWW Comber H Cancer incidence and mortality patterns in Europe: estimates for 40 countries in 2012. Eur J Cancer (2013) 49:1374–403. 10.1016/j.ejca.2012.12.027 23485231

[5] Ferlay J Colombet M Soerjomataram I Dyba T Randi G Bettio M Cancer incidence and mortality patterns in Europe: estimates for 40 countries and 25 major cancers in 2018. Eur J Cancer (2018) 103:356–87. 10.1016/j.ejca.2018.07.005 30100160

[6] Weber A Mery L Nagy P Polgar C Bray F Kenessey I . Evaluation of data quality at the Hungarian national cancer registry, 2000-2019. Cancer Epidemiol (2023) 82:102306. 10.1016/j.canep.2022.102306 36521336

[7] NEAK. Nemzeti Egészségbiztosítási Alapkezelő [National Health Insurance Fund of Hungary]. Annual Statistical Report 2022 (2023). Available online at: https://www.neak.gov.hu/oldalak/nyelvi-oldalak/english (Accessed November 20, 2024).

[8] Bogos K Kiss Z Gálffy G Tamási L Ostoros G Müller V Revising incidence and mortality of lung cancer in central Europe: an epidemiology review from Hungary. Front Oncol (2019) 9:1051. 10.3389/fonc.2019.01051 31709174 PMC6819432

[9] International Agency for Research on Cancer. Globocan 2020: Hungary Fact Sheet. Lyon: IARC (2021). Available online at: https://gco.iarc.who.int/media/globocan/factsheets/populations/348-hungary-fact-sheet.pdf (Accessed December 4, 2024).

[10] Heyzine. Korányi bulletin (2023). Available online at: https://heyzine.com/flip-book/d280e3fd6d.html (Accessed December 4, 2024).

[11] Siegel R Miller K Fuchs H Jemal A . Cancer statistics. CA Cancer J Clin (2022) 72(1):7–33. 10.3322/caac.21708 35020204

[12] Dingemans A-MC Früh M Ardizzoni A Besse B Faivre-Finn C Hendriks LE On behalf of the ESMO guidelines committee* small-cell lung cancer (SCLC): ESMO clinical practice guidelines for diagnosis, treatment and follow-up. Ann Oncol (2021) 32(7):839–53. 10.1016/j.annonc.2021.03.207 33864941 PMC9464246

[13] Meerbeeck JP Fennell DA Ruysscher DKD . Small-cell lung cancer. The Lancet (2011) 378(9804):1741–55. 10.1016/S0140-6736(11)60165-7 21565397

[14] Früh M Ruysscher DD Popat S Crinò L Peters S Felip E . Small-cell lung cancer (SCLC): ESMO clinical practice guidelines for diagnosis, treatment and follow-up†. Ann Oncol (2013) 24:vi99–105. 10.1093/annonc/mdt178 23813929

[15] Blackhall F Girard N Livartowski A McDonald L Roset M Lara N Treatment patterns and outcomes among patients with small-cell lung cancer (SCLC) in Europe: a retrospective cohort study. BMJ Open (2023) 13(2):e052556. 10.1136/bmjopen-2021-052556 36746549 PMC9906168

[16] Paz-Ares L Dvorkin M Chen Y Reinmuth N Hotta K Trukhin D Durvalumab plus platinum–etoposide *versus* platinum–etoposide in first-line treatment of extensive-stage small-cell lung cancer (CASPIAN): a randomised, controlled, open-label, phase 3 trial. The Lancet (2019) 394(10212):1929–39. 10.1016/S0140-6736(19)32222-6 31590988

[17] Horn L Mansfield AS Szczęsna A Havel L Krzakowski M Hochmair MJ First-line atezolizumab plus chemotherapy in extensive-stage small-cell lung cancer. N Engl J Med (2018) 379(23):2220–9. 10.1056/NEJMoa1809064 30280641

[18] Gálffy G Hécz R Bujdosó R Gáspár E Korompay R Hoffer J Early-stage resectable non-small cell lung cancer in Hungary. Pathol Oncol Res (2025) 31:1612152. 10.3389/pore.2025.1612152 40791752 PMC12336066

[19] Ganti AKP Loo BW Bassetti M Blakely C Chiang A D’Amico TA Small cell lung cancer, version 2.2022, NCCN clinical practice guidelines in oncology. J Natl Compr Canc Netw (2021) 19(12):1441–1464. 10.6004/jnccn.2021.0058 34902832 PMC10203822

[20] O’Sullivan DE Cheung WY Syed IA Moldaver D Shanahan MK Bebb DG Real-world treatment patterns, clinical outcomes, and health care resource utilization in extensive-stage small cell lung cancer in Canada. Curr Oncol (2021) 28(4):3091–103. 10.3390/curroncol28040270 34436036 PMC8395392

[21] Reguart N Pérol M Cortinovis D Puntis S Öhrling K Archangelidi O A cross-sectional analysis of treatment patterns in small-cell lung cancer in five European countries. Future Oncol (2025) 20(17):1151–64. 10.2217/fon-2022-1315 38031886 PMC11318697

[22] Alhattab RAN McKinley JM Hunter RF Delargy CM Wallace SM Bennett D Lung cancer burden attributable to ambient particulate matter: a nationally representative population-based case-control study. Br J Cancer (2025) 133(12):1872–9. 10.1038/s41416-025-03207-x 41053161 PMC12689774

[23] Field RW Withers BL . Occupational and environmental causes of lung cancer. Clin Chest Med (2012) 33(4):681–703. 10.1016/j.ccm.2012.07.001 23153609 PMC3875302

[24] Darby S Hill D Auvinen A Barros-Dios JM Baysson H Bochicchio F Radon in homes and risk of lung cancer: collaborative analysis of individual data from 13 European case-control studies. BMJ (2005) 330(7485):223. 10.1136/bmj.38308.477650.63 15613366 PMC546066

[25] Evans WK Shepherd FA Feld R Osoba D Dang P Deboer G . VP-16 and cisplatin as first-line therapy for small-cell lung cancer. J Clin Oncol (1985) 3(11):1471–7. 10.1200/JCO.1985.3.11.1471 2997406

[26] Roth BJ Johnson DH Einhorn LH Schacter LP Cherng NC Cohen HJ Randomized study of cyclophosphamide, doxorubicin, and vincristine versus etoposide and cisplatin versus alternation of these two regimens in extensive small-cell lung cancer: a phase III trial of the southeastern cancer study group. J Clin Oncol (1992) 10(2):282–91. 10.1200/JCO.1992.10.2.282 1310103

[27] Azar I Yazdanpanah O Jang H Austin A Kim S Chi J Comparison of carboplatin with cisplatin in small cell lung cancer in US veterans. JAMA Netw Open (2022) 5(10):e2237699. 10.1001/jamanetworkopen.2022.37699 36264573 PMC9585434

[28] Damiano P Stefani A Avancini A Belluomini L Bria E Pilotto S . Real-world evidence in extensive disease small cell lung cancer: the missing piece of the puzzle. Crit Rev Oncol Hematol (2025) 207:104618. 10.1016/j.critrevonc.2025.104618 39827977

[29] Andreas V Faltys M Alexander M Rogers J Parakh S Bowyer S Second-line treatment strategies and clinical outcomes after progression on chemoimmunotherapy in extensive-stage small-cell lung cancer. Lung Cancer (2025) 209:108786. 10.1016/j.lungcan.2025.108786 41077000

[30] (II. 28.) NEFMI rendelet az egészségbiztosítási alapból a 959A–L, valamint 9511–9515 homogén betegségcsoportok szerint finanszírozott daganatellenes terápiákról. (2012). Available online at: https://net.jogtar.hu/jogszabaly?docid=a1200011.nem#lbj7ide4d (Accessed June 16, 2026).

[31] Valette CA Filleron T Debieuvre D Lena H Pérol M Chouaid C Treatment patterns and clinical outcomes of extensive stage small cell lung cancer (SCLC) in the real-world evidence ESME cohort before the era of immunotherapy. Respir Med Res (2023) 84:101012. 10.1016/j.resmer.2023.101012 37307617

[32] EMA. (2025). Available online at: https://www.ema.europa.eu/, (Accessed April 18, 2026).

