# Supplementary figures and images for: Extensive-stage small cell lung cancer in Hungary: a real-world analysis (2013–2022)
Source: Pathol Oncol Res. 2026 Jul 16;32:1612491. doi: 10.3389/pore.2026.1612491 (PMC13422220; doi:10.3389/pore.2026.1612491)

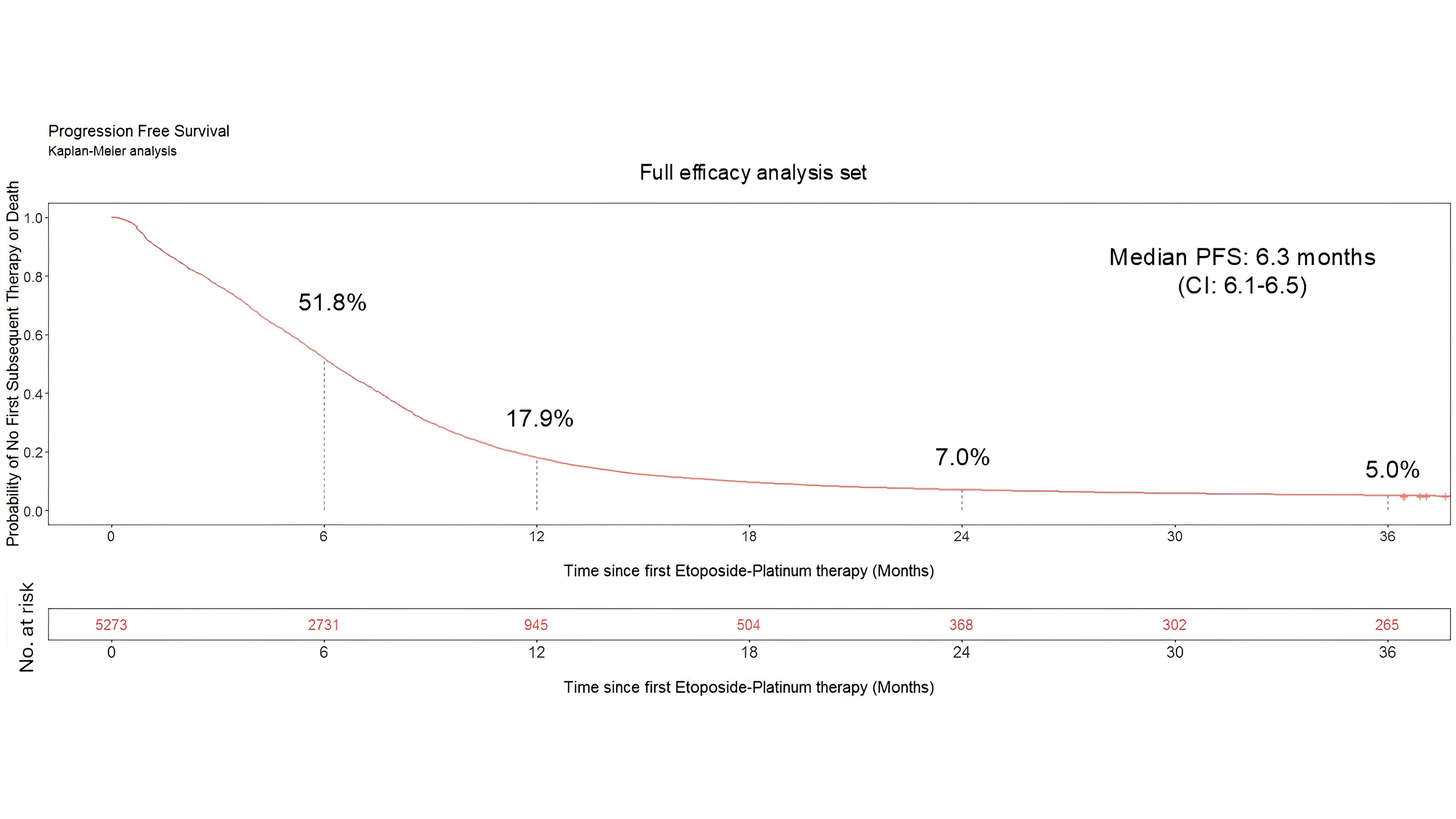

Supplement: Supplementary file 1 [file Image3.tiff]

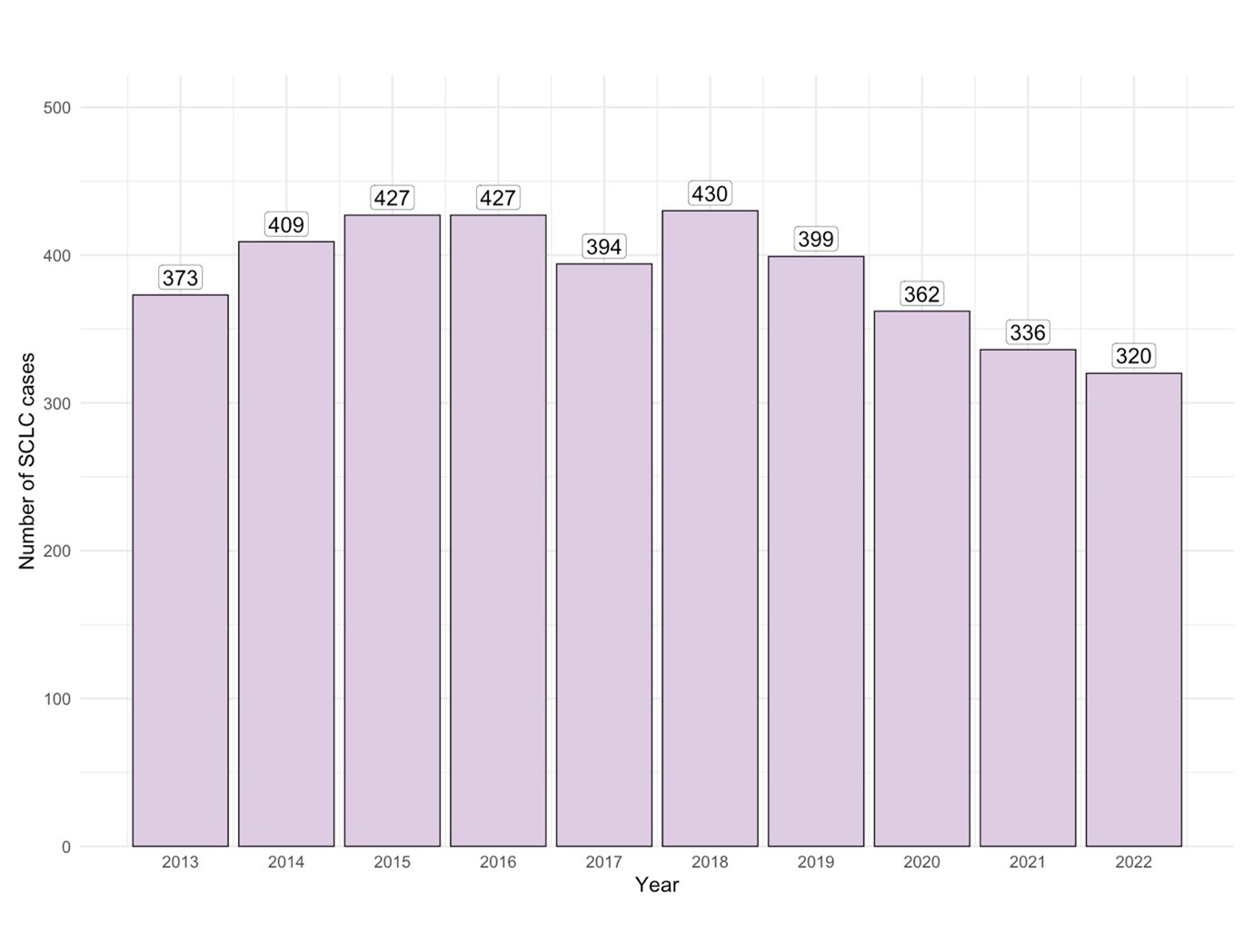

Supplement: Supplementary file 2 [file Image1.JPEG]

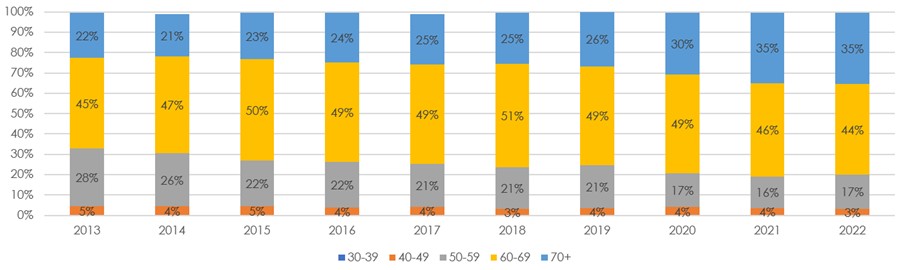

Supplement: Supplementary file 3 [file Image2.JPEG]

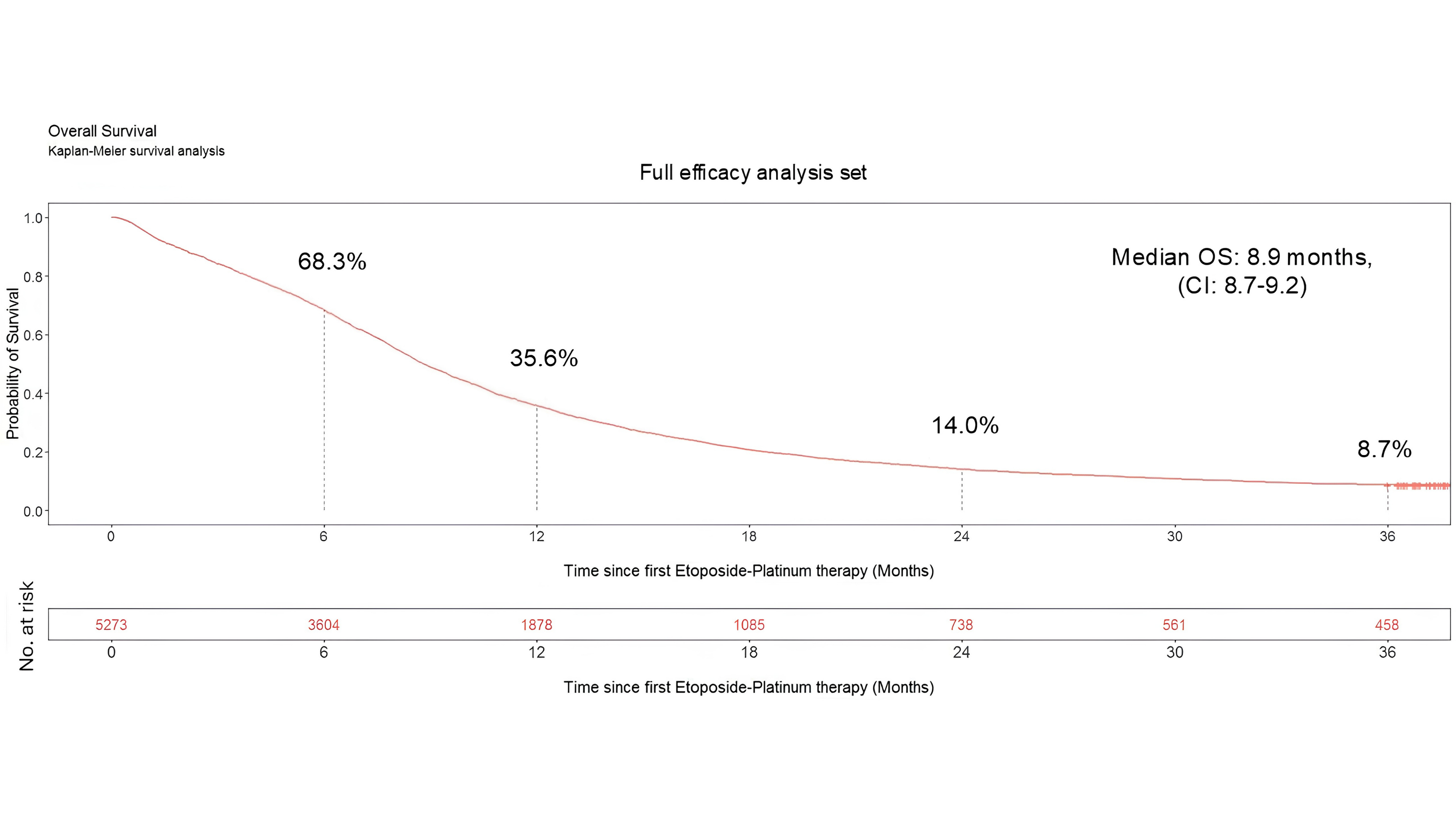

Supplement: Supplementary file 4 [file Image4.tiff]
